# Morphological changes of the root apex in anterior teeth with periapical periodontitis: an in-vivo study

**DOI:** 10.1186/s12903-022-02062-z

**Published:** 2022-02-05

**Authors:** Chen-chen Zhang, Ya-jing Liu, Wei-dong Yang, Qian-nan Zhang, Ming-zhu Zha, Shan-hui Wen, Qi Wang

**Affiliations:** 1 Department of Endodontics, Nanjing Stomatological Hospital, Medical School of Nanjing University, Zhongyang Road 30, Nanjing City, 210008 Jiangsu Province China; 2 Department of Dentomaxillofacial Radiology, Nanjing Stomatological Hospital, Medical School of Nanjing University, Nanjing, 210008 Jiangsu China

**Keywords:** Periapical periodontitis, Anterior teeth, Cone-beam computed tomography, 3D reconstruction, Root apex morphology

## Abstract

**Introduction:**

The aim was to analyze the morphological changes of root apex in anterior teeth with periapical periodontitis.

**Methods:**

32 untreated anterior teeth with periapical periodontitis were enrolled, compared with the healthy contralateral teeth. Two-dimensional measurement of Cone-beam computed tomography was used to determine the location and measure diameter of the apical constriction according to Schell’s methods. An open-source software (3D Slicer) was used to reconstruct the teeth. The apical constriction form was analysis according to Schell’s topography. The distances of apical constriction to apical foramen and anatomical apex were measured respectively.

**Results:**

The difference value between buccolingual and mesiodistal diameter was (0.06 ± 0.09) mm and (0.04 ± 0.04) mm in periapical periodontitis and controls (*p* < 0.05). The mean distance between apical constriction and anatomical apex was significantly shorter in periapical periodontitis than controls, so was the mean distance of apical constriction to apical foramen. The most common form of apical constriction was flaring (65.6%) in periapical periodontitis.

**Conclusions:**

The anterior teeth with periapical periodontitis had shorter distances of apical constriction to anatomical apex and apical foramen, bigger disparities between the diameters of buccolingual and mesiodistal, and higher proportion of flaring apical constriction.

**Supplementary Information:**

The online version contains supplementary material available at 10.1186/s12903-022-02062-z.

## Introduction

Periapical periodontitis (AP) is one of the most common dental diseases in clinical work [1, 2]. It could cause periapical tissues destruction, hard tissue resorption, and local inflammation. The main clinical symptoms are swelling, pain, tenderness and sinus tract formation. Increased inflammation may promote more obvious bone resorption and the formation of radicular cysts [3]. If an apical periodontitis developed into a cyst, root canal therapy might not be sufficient. Sometimes, apicectomy or even extraction of the affected teeth could be required [4]. On the other hand, recent studies have found that AP was associated with cardiovascular diseases, diabetes mellitus and coronary heart disease [5, 6]. Numerous epidemiological studies have investigated the incidence of AP, ranged from 27 to 64% [2, 7, 8].

As the end of the root canal, apical foramen communicates with the periapical tissue and opens onto the root surface. It is reported that apical foramen could be the passageway of infections to enter or exit the root canal [9]. And the root canal with irregularly-shaped apical foramen are difficult to be filled completely [9]. Therefore, apical morphology was considered and proved to be a significant factor related to the clinic curative effect of root canal treatment [10].

Using scanning electron microscopy (SEM) to examine the extracted root apices of teeth with AP, Furusawa and Asai [11] observed resorptions around apical foramen. Moreover, their results demonstrated that 80% of teeth with AP displayed a wide opening apical foramen. External apical root resorptions around the apical foramen were also observed with SEM in AP [12]. These studies suggested that AP could lead to changes and destruction around the apical foramen.

Although researches achieved a better understanding of influences of AP on the morphology at the root apex, these studies focused on ultrastructure of apical surfaces using scanning electron microscopy [12] or micro-computed tomography (micro-CT) [13, 14]. Both of them provide high resolution owing to the high energy parameters and smaller voxel sizes, but require high radiation doses and long scanning time, which makes them limited to laboratory studies. So far, there were no in-vivo studies focusing on the changes of apical morphology in AP. Cone-beam computed tomography (CBCT) scanning which we used in the present study is a noninvasive method directly used on patients.

The present study aimed to analysis the changes of the diameter of apical constriction, the distance of apical constriction to apical foramen, distance of apical constriction to anatomical apex, and the apical constriction form in anterior teeth with AP in a Chinese sub-population, in order to provide a new insight into the relationship of AP and the morphology of root apex.

## Materials and methods

### Sample selection

Patients visited Nanjing Stomatological Hospital, Medical school of Nanjing University between January 2018 and February 2019 and underwent CBCT for reasons independent of the present study, including orthodontic treatment or implants unrelated to the present study. CBCTs were also performed independent of this study. The inclusion criteria were: adult patients; anterior teeth with AP (radiographically by CBCT images) and the contralateral homonymous teeth without AP; single root canal; without root canal treatment, post, or crowns; no calcified root canals; a fully formed apex. Through case history inquiry, the patients with the history of developmental disorders, systemic disease, malignancy, orthodontic treatment and trauma were excluded. Only high-resolution images were included to ensure analytic accuracy.

To perform sample size calculation, the distances between AC-AA and AC-AF in anterior teeth were measured in a pilot study with seven subjects. According to the data of the pilot study, a sample size of 6 could achieve 80% power by the two-sided paired t-test carried out by PASS software. By screening cases, we collected 32 cases meeting the inclusion criteria. Although a sample size of 6 could achieve statistical significance, we enrolled all cases in order to make the results more reliable.

Finally, the study consisted of 32 patients (18 females and 14 males), with a mean ± SD age of 38.0 ± 11.5 years. The patients enrolled were Chinese born in China. All patient-related materials and data were stored anonymously and only made available to the study investigators.

### Image acquisition

All CBCT images were obtained by a NewTom VG scanner (QR srl, Verona, Italy). The voxel size was set 0.125 mm and operated according to the manufacturer’s instructions. All CBCT exposures were performed by an experienced licensed radiologist with the minimum exposure necessary for adequate image quality, following strictly the as low as reasonably achievable protocol.

The images were exported in Digital Imaging and Communications in Medicine (DICOM) data format. Then the data were imported into 3D Slicer 4.8.1 (https://www.slicer.org/), a free open-source software for medical image processing [15], analyzed using a 13.9-in. HUAWEI Mate Book X Pro 2020 (HUAWEI Corporation, Shenzhen, China) screen with a resolution of 3000 * 2000 pixels in a darkroom.

### Two-dimensional (axial slices) measurement

**Fig. 1:**
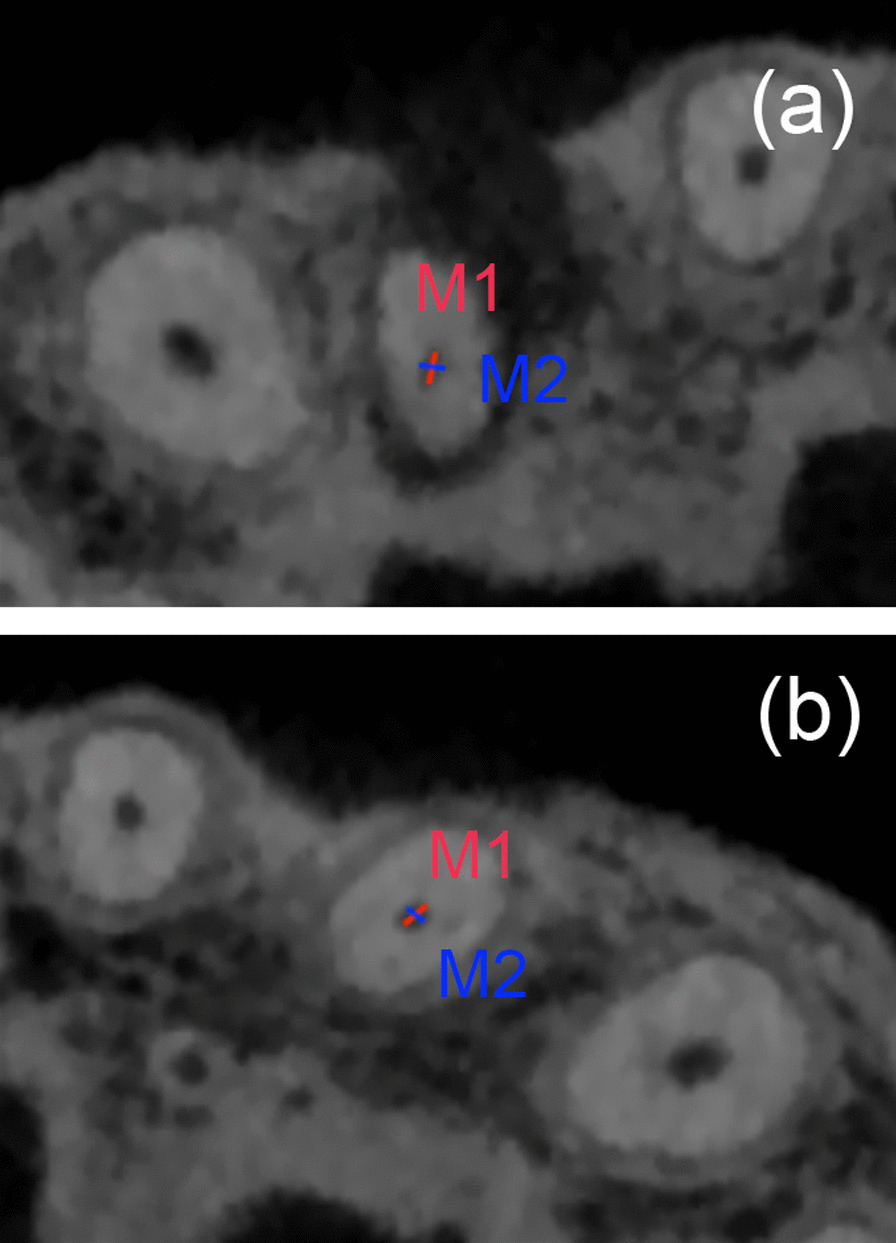
The measurement of apical constriction diameters in CBCT images. Horizontal plane at the apical constriction level of the root canal with AP (**a**) and without AP (**b**) were measured respectively. The diameter was determined by drawing a line between the two most distant pixels of the root canal walls. M1 represented the diameter of BL (red line), while M2 represented the diameter of MD (blue line)

The apical constriction was defined to be the apical cross sections having the smallest area [16]. According to Schell’s methods [17] of determining the location of apical constriction, CBCT analysis of serial cross sections, perpendicular to the canal axis, were analyzed from both buccolingual (BL) and mesiodistal (MD) aspects. The root canal measurements included BL and MD diameters from the apex. CBCT images were carefully examined from the pulp chamber to the apical apex by continuously scrolling the toolbar on axial view to evaluate the topography of the apical constriction. By rolling the middle axis of the mouse, the grayscale value was continuously changed, until the best display was obtained. On the axial view, the diameters of BL and MD were measured (Fig. 1), and the difference value of diameter between BL and MD was calculated. According to the diameter of BL and MD, two operators determine the location of the apical constriction individually, until a consensus was reached.

### 3D reconstruction and measurement

Each structure including root and canal was assigned a label. By using the “Editor” module of 3D Slicer software tools, the contour of tooth image (root canal and root) was segmented semi-automatically or manually slice by slice, with a mouse. The segmentations were mainly performed on axial slices. In addition, some adjustments were then performed on sagittal and coronal views. By using the image processing tool in the software, the brightness and contrast were adjusted to achieve optimum visualization. Then the anatomical structures (roots and canals) were reconstructed using the “Merge And Build” module of 3D Slicer. Similarly, the optimal reconstruction was ensured by adjusting the opacity of the images.

**Fig. 2:**
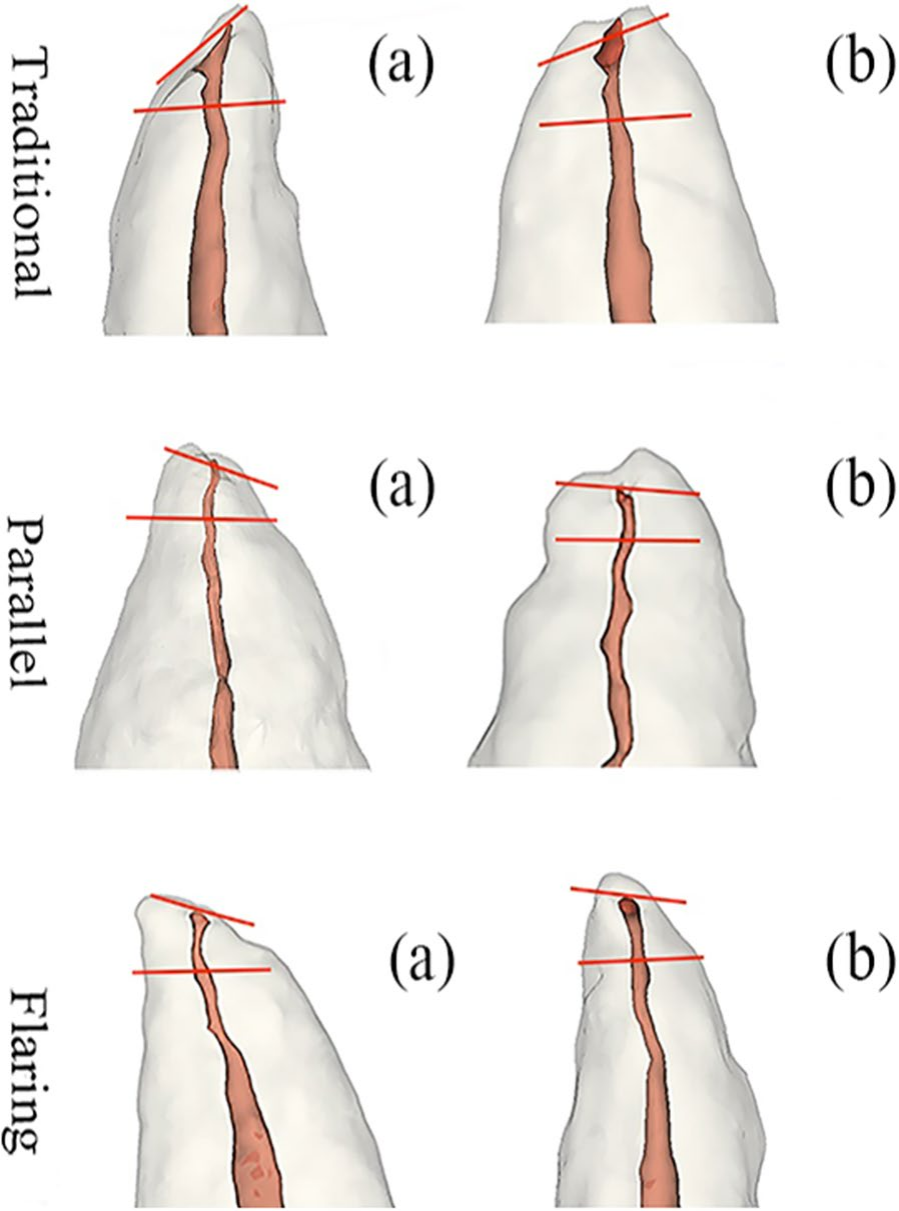
The longitudinal perspective view of reconstructed teeth. **a**, **b** The mesiodistal views and buccolingual views respectively. Horizontal red lines were portrayed in the apical region

In this study, according to the topography of the apical constriction of Schell [17], the parallel form was recognized when the minor diameter of the canal extended steadily for a long distance (> 2.5 mm) and only widened for a short distance (0.1 mm) at the foramen. When the canal walls widened apically and coronally from the narrowest zone, it was considered to be traditional. In the flaring form, the canal walls constantly diverged towards the apex. In order to eliminate the subjective and objective factors of the cutting process on the outcomes, we chose to use the longitudinal 3D model as a whole for analysis**.** Three forms of constriction morphologies were observed in the mesiodistal views and the buccal-lingual views of the 3D models (Fig. 2).

Horizontal red lines in Fig. 2 were portrayed in the apical region (approximately 1–3 mm) where a constriction might be present. The apical morphology between the red lines was examined and categorized by evaluators in the longitudinal view of reconstructed teeth. The constriction forms were subdivided into traditional, parallel and flaring.

**Fig. 3:**
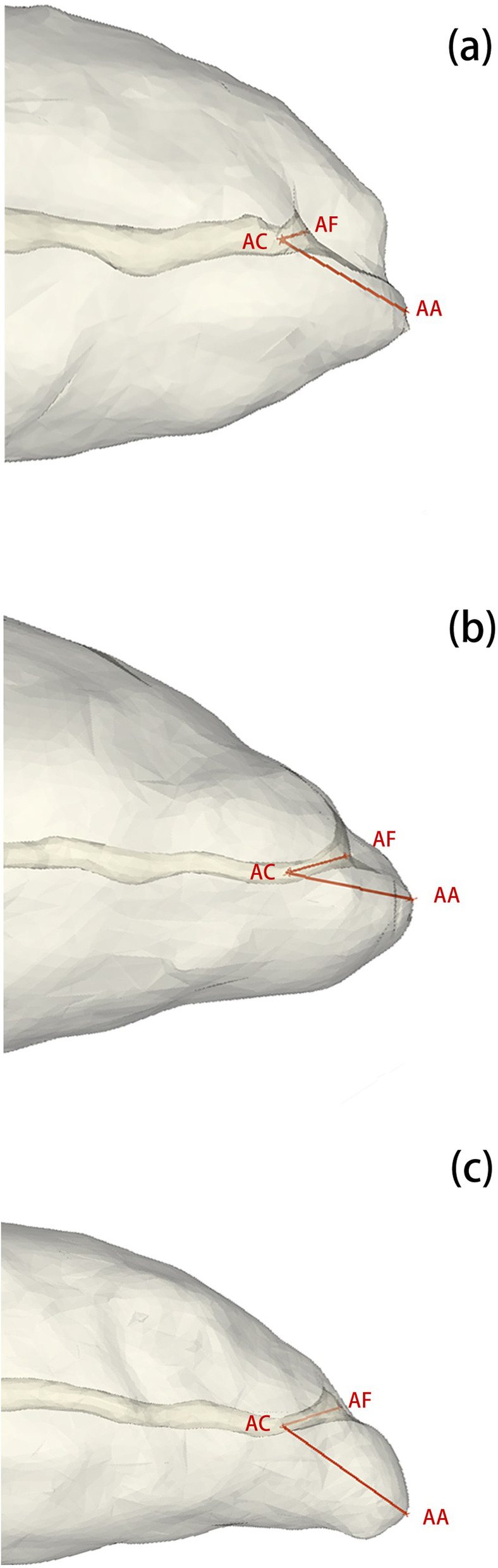
Measurements of different apical constriction forms. **a** The measurement of traditional form, while **b**, **c** were parallel and flaring forms respectively

In the 3D model of teeth, the distances from apical constriction to foramen (AC-AF) and to anatomical apex (AC-AA) of each canal were recorded. On 3D views, the distance of AC-AF and AC-AA were measured directly (Fig. 3).

### Radiologic evaluation

To check for the inter- and intra-observer reliabilities, manual segmentations were performed twice for each of 10 teeth which were not included in this study by two independent examiners, with a 1-week interval between the assessments. The inter-examiner agreement value was 0.854. The intra-examiner agreement values were 0.878 and 0.889 respectively for the first and second assessments.

### Statistical analysis

Data from the evaluators were collected and analyzed. Percentages and frequencies were used to describe categorical variables. Quantitative data from measurements were presented as mean and standard deviation. Comparisons were made between AP teeth and healthy teeth data (AC-AA, AC-AF) through paired *t* tests with a significance level of 5%. Differences in constriction forms were analyzed by Fisher exact test or Chi square test according to gender. Statistical Package for Social Sciences 25.0 (IBM Co, New York, NY) was used for statistical analysis. The significance level was set at *p* < 0.05.

### Ethical approval and consent to participate

All stages of the investigation were conducted in accordance with the principles of tne Helsinki Declaration. The Research Ethics Committee of Nanjing Stomatological Hospital, Medical school of Nanjing University (NO: JX-2020NL-013,the date of ethical approval: 12 November 2020) reviewed and approved the study protocol. Ethical considerations were considered during each step of the research process. Informed consent was obtained from all patients.

## Results

In this retrospective study, a total of 64 teeth in 32 patients were analyzed. According to the diameter of BL and MD, the apical constriction was located in each tooth. The diameters of BL and MD at apical constriction were shown in Table 1. The mean BL diameter of apical constrictions was significantly longer in anterior teeth with AP than healthy teeth (*p* < 0.05). The mean MD diameters of apical constriction was longer in anterior teeth with AP than healthy teeth, without significantly difference (*p* > 0.05). The difference value between BL and MD diameter was significantly bigger in anterior teeth with AP than healthy teeth (*p* < 0.05).

**Table 1:** The difference value between BL and MD diameters (mm) at apical constriction

Group	BL diameter	MD diameter	BL-MD
	Mean ± SD	Mean ± SD	Mean ± SD
AP	0.32 ± 0.10*	0.26 ± 0.06	0.06 ± 0.09*
Healthy teeth	0.28 ± 0.08	0.24 ± 0.07	0.04 ± 0.04

*SD* standard deviation

*p < 0.05, paired *t* tests

**Fig. 4:**
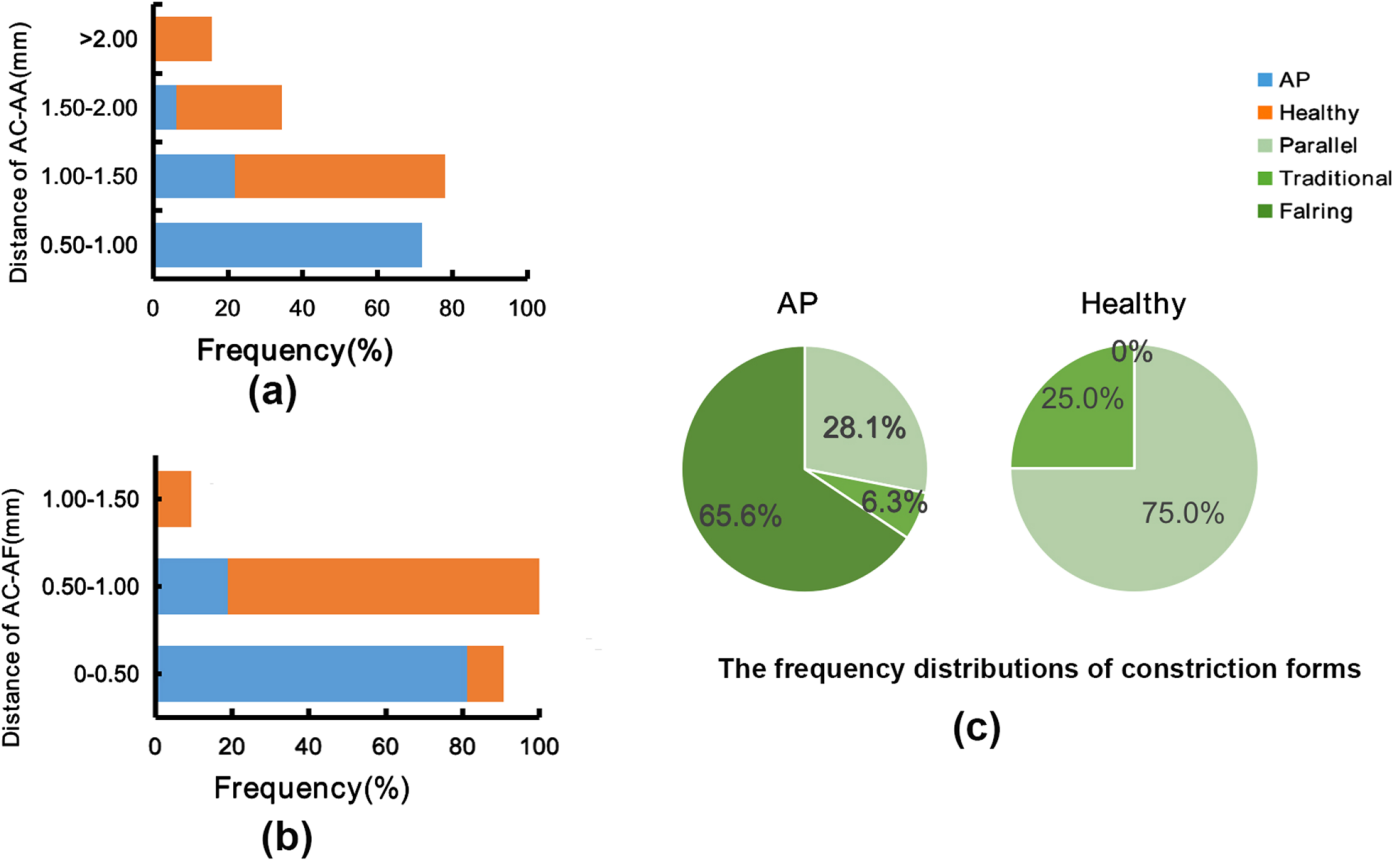
The distributions of AC-AA, AC-AF and constriction forms in periapical periodontitis and healthy teeth. **a**, **b** The distances and distributions of AC-AA and AC-AF in periapical periodontitis and healthy teeth. **c** The proportions of different constriction forms in teeth with periapical periodontitis and healthy ones

By the longitudinal perspective view of 3D reconstructed teeth, the constriction forms of apical areas were analyzed (Table 2). The anterior teeth with AP had higher proportion of flaring apical constriction. Significant differences were not found between sexes (*p* > 0.05) (Table 2). The distributions of the constriction forms in AP and healthy teeth were shown in Fig. 4c. Among the three constriction forms, the flaring constrictions were only found in the teeth with AP.

**Table 2:** The distributions of constriction forms in  AP and healthy teeth

Gender	AP (N, %)			Healthy teeth (N, %)			Total
	Parallel	Traditional	Flaring	Parallel	Traditional	Flaring	
Male	3 (4.7)	–	11 (17.2)	10 (15.6)	4 (6.25)	–	28 (43.8)
Female	6 (9.4)	2 (3.1)	10 (15.6)	14 (21.9)	4 (6.25)	–	36 (56.3)
Total	9 (14.1)	2 (3.1)	21 (32.8)	24 (37.5)	8 (12.5)	–	64 (100)

*p < 0.05, Fisher exact test

Measurements of distances between AC to AA and AF were depicted in Table 3 and Fig. 4. The anterior teeth with AP had significantly shorter distances of apical constriction to anatomical apex and apical foramen (*p* < 0.05).

**Table 3:** Distances of AC-AA and AC-AF (mm) in  AP and healthy teeth

Group	AC-AA	AC-AF
	Mean ± SD	Mean ± SD
AP	0.97 ± 0.25*	0.39 ± 0.12*
Healthy teeth	1.59 ± 0.48	0.70 ± 0.18

*SD* standard deviation

*p < 0.05, paired *t* tests

## Discussion

The present study provided a detailed anatomical description of morphological changes of the root apex in anterior teeth with AP based on a retrospective analysis of CBCT images. Numerous methods could be used to evaluate morphology of root apex, such as intraoral periapical radiograph [18], longitudinal cutting [19], stereomicroscope [20], micro-computed tomography [13, 17, 21–25] and CBCT [26]. Among these methods, CBCT and periapical radiography meet both non-invasive requirements and in-vivo conditions. Various studies have shown that CBCT is much more reliable than periapical radiography in diagnosing complex root canal morphology, because it could provide three-dimensional images of three-dimensional objects [27]. Moreover, CBCT has been proved to be more sensitive and accurate to identify apical periodontitis than the two-dimensional imaging technique [28, 29]. In the accuracy of detection of the root canal configuration, CBCT imaging has been showed no difference with the gold standard (micro-CT) [30].

In our study, an apical constriction was found in all the root canals examined. The results were similar to the researches of Schell et al. [17] and Dummer et al. [31]. However in the studies of Meder-Cowherd et al. [25], Marceliano-Alves et al. [23] and Wu et al. [32], apical constriction was not identified in every root. The reason may be that two different longitudinal sections (buccal/lingual or mesial/distal view) of the same canal showed different topographical canal outlines, so longitudinal section could not show the narrowest part of the canal [24]. In many canals, the narrowest part only could be detected by analyzing the cross-sectional areas along the path of the root canal.

It is usually considered that the ideal apical end point of root canal therapy was the apical constriction [16]. The size of the root canal instruments was classified by the size of the apical constriction, which was determined by the minimal area of root canal. In most canals, the instrument size was determined by the maximum diameter. In the present study, the disparity between BL and MD diameter was larger in the teeth with AP (*p* < 0.05) (Table 3). Because of the difference, the instrument size that would fit the apical constrictions of teeth with chronic AP may be larger than teeth without apical periodontitis.

In the present study, the most common form of the constriction was flaring (65.6%) in teeth with AP, while the most common form was parallel (75.0%) in healthy teeth (Table 2). Dummer et al. [31] inspected longitudinal sections and found that the most frequent form of the constriction was the traditional form (46.0%). Meder-Cowherd et al. [25] found that the most frequent form of the constriction in teeth was the parallel form (35.0%). This discrepancy may due to the differences in tooth forms. Dummer studied central incisor, lateral incisors, canines and premolars. In Meder’s research, they studied palatal roots of maxillary molars. While in our study, the samples were anterior teeth including central incisors, lateral incisor and canines. What’s more, the instruments used to detect the constriction were different. Dummer used the microscopic and Meder used micro-CT, while we made use of CBCT in the present study (Additional file 1).

In our study, the distances of AC-AF and AC-AA were different from some researches. In Wolf’s study [14], the mean distances between AC and AA were 0.82 mm (MB), 0.81 mm (DB) and 1.02 mm (P) in maxillary first molars, 0.54 mm (MB), 0.43 mm (DB) and 0.63 mm (P) in maxillary second molars. In Mousavi’s study under microscopic, the mean distance between AC and AF was 0.85 ± 0.33 mm in incisors, and the mean distance between AC and AA was 1.23 ± 0.39 mm in incisors [33]. By the use of microscopic Kutter and Dummer found that the narrowest part of the canal was 0.59 mm to AF [16] or 0.89 mm to AA on average [31]. Several reasons may account for the differences. First, the methods were different. Our study was a three-dimensional reconstruction analysis, while other researchers mentioned above conducted only two-dimensional analysis. Second, the samples in the studies mentioned above were extracted teeth. In contrast, the samples in this study were in vivo, which would represent the actual situation better. Third, the ethnic population in this study was different from that in other studies mentioned above. Mousavi studied the Iranian population, while the studies of Wolf and other researches did not account for it. These reasons may result in different distances of AC-AF and AC-AA. In canals without apical periodontitis, the apical limit for root canal instrumentation and filling is determined by the apical constriction and the apical foramen.

In summary, the present retrospective study provided evidences to support that the teeth with AP had shorter distances of AC-AA and AC-AF, bigger disparities between the diameters of BL and MD, and higher proportion of flaring apical constriction. These data may help clinicians to understand the morphological changes of the root apex in anterior teeth with AP and provide references for clinical treatment.

## Conclusions

The anterior teeth with periapical periodontitis had shorter distances of apical constriction to anatomical apex and apical foramen, bigger disparities between the diameters of buccolingual and mesiodistal, and higher proportion of flaring apical constriction.

## Supplementary Information

**Additional file 1**.** Table S1** showed the raw data of this study, including the sex and age of patients, the diameters of constrictions, the distances of AC-AF and AC-AA, and forms of constrictions.

## Data Availability

The datasets used and/or analysed during the current study are available from the corresponding author on reasonable request. All data generated or analysed during this study are included in its supplementary information files.
